# Early Detection of *Legionella pneumophila* and *Aspergillus* by mNGS in a Critically Ill Patient With *Legionella* Pneumonia After Extracorporeal Membrane Oxygenation Treatment: Case Report and Literature Review

**DOI:** 10.3389/fmed.2021.686512

**Published:** 2021-06-30

**Authors:** Ruiming Yue, Xiaoxiao Wu, Tianlong Li, Li Chang, Xiaobo Huang, Lingai Pan

**Affiliations:** ^1^Department of Critical Care Medicine, Sichuan Provincial People's Hospital, University of Electronic Science and Technology of China, Chengdu, China; ^2^Chinese Academy of Sciences Sichuan Translational Medicine Research Hospital, Chengdu, China

**Keywords:** *Legionella*, invasive pulmonary aspergillosis, mNGS, ECMO, diagnosis

## Abstract

*Legionella pneumophila* can cause pneumonia, leading to severe acute respiratory distress syndrome (ARDS). Because of its harsh growth requirements, limited detection methods, and non-specific clinical manifestations, diagnosing *Legionella* pneumonia remains still challenging. Metagenomic next-generation sequencing (mNGS) technology has increased the rate of detection of *Legionella*. This study describes a patient who rapidly progressed to severe ARDS during the early stage of infection and was treated with extracorporeal membrane oxygenation (ECMO). Although his bronchoalveolar lavage fluid (BALF) was negative for infection and his serum was negative for anti-*Legionella* antibody, mNGS of his BALF and blood showed only the presence of *Legionella pneumophila* (blood mNGS reads 229, BALF reads 656). After antibiotic treatment and weaning from ECMO, however, he developed a secondary *Aspergillus* and *Klebsiella pneumoniae* infection as shown by mNGS. Mechanical ventilation and antibiotic treatment were effective. A search of PubMed showed few reports of secondary *Aspergillus* infections after *Legionella* infection. Severe pneumonia caused by any type of pathogenic bacteria may be followed by *Aspergillus* infection, sometimes during extremely early stages of infection. Patients with severe pneumonia caused by *Legionella* infection should undergo early screening for secondary infections using methods such as mNGS, enabling early and precise treatment, thereby simplifying the use of antibiotics and improving patient prognosis.

## Introduction

*Legionella pneumophila* is a common atypical pathogen that can cause pneumonia, and is thought to be responsible for about 2–15% of patients with community-acquired pneumonia who require hospitalization (1, 2). *Legionella* pneumonia can rapidly lead to the development of acute respiratory distress syndrome (ARDS) and multiple organ dysfunction syndrome (MODS) (3).

*Legionella* bacteria often grow and multiply in water. Patients with *Legionella* pneumonia often have a non-specific epidemiological history (e.g., bathing, travel history, and cruises) and clinical symptoms, and laboratory tests lack specificity. These patients can present with slow pulse, gastrointestinal symptoms, and/or muscle pain, as well as with involvement of the kidneys and/or nervous system (4).

Traditional methods of culturing *Legionella* are often time-consuming, and cultures are easily contaminated. Moreover, harsh culture conditions are required, further decreasing the positivity rate of traditional cultures (5). Clinically, *Legionella* is often diagnosed by the presence of anti-*Legionella* antibodies.

Most patients, however, develop anti-*Legionella* antibodies around 3 weeks after the onset of the disease, resulting in delayed diagnosis (4). The emergence of metagenomic next-generation sequencing (mNGS) has markedly improved the rate of early diagnosis of *Legionella* pneumonia. Although extracorporeal membrane oxygenation (ECMO) treatment has increased the survival rate of patients infected with *Legionella* bacteria, nosocomial infections and secondary fungal infections are common and serious complications of ECMO treatment. *Aspergillus* infection has been reported after ECMO treatment, especially secondary pulmonary *Aspergillus* infection in patients with severe influenza or COVID-19, and in immunosuppressed patients (6–10). Few reports, however, have described patients with *Legionella* pneumonia co-infected with invasive pulmonary aspergillosis (IPA). The present report describes a patient with early IPA and sensitive *Klebsiella pneumoniae* secondary to *Legionella pneumophila* serogroup one infection after ECMO treatment. To our knowledge, this report is the first to describe a patient with severe *Legionella* pneumonia treated by venous-venous ECMO (VV-ECMO) who developed a secondary IPA infection after the first stage of improvement. This study showed that mNGS technology can improve the early detection and treatment of *Legionella* pneumonia and secondary Aspergillus infection.

## Case Presentation

**Figure 1:**
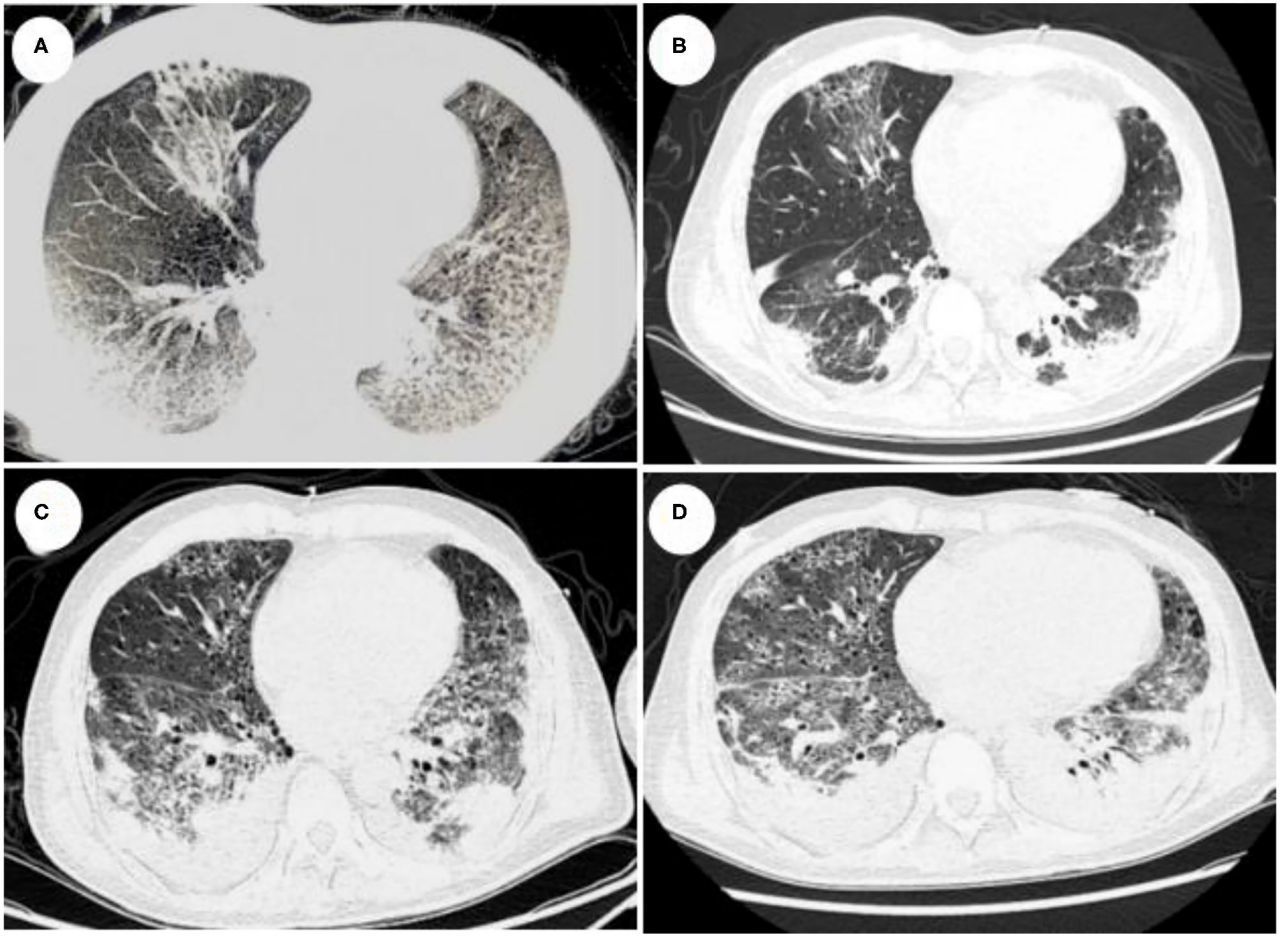
**(A)** CT scan taken on February 5 (day 2), showing multiple infectious foci and interstitial lesions in the right lung, the lingual segment of the upper lobe of the left lung, and the subpleura of the lower lobe of the left lung. **(B)** CT scan taken on February 19 (day 16), 7 days after the patient was weaned from ECMO, showing scattered patchy shadows and meshwork blurred shadows in both lungs, as well as some consolidation, indicative of significant improvements. **(C)** CT scan taken on February 24 (day 21), after the patient again presented with dyspnea and was re-admitted to the ICU. The CT scan showed patches in lobular segments of both lungs, honeycomb-like changes, partial consolidation, and lesion expansion. **(D)** CT scan taken on March 01 (day 26), showing patchy, gridded, cellulite-like changes, with bronchiectasis in multiple lobes of the lungs.

A 46-year-old man was admitted to the intensive care unit (ICU) on February 4, 2021, after experiencing fever (highest temperature 39.0°C), accompanied by chills and diarrhea with yellow watery stool, for 2 days and dyspnea for 1 day. A chest computed tomography (CT) scan showed multiple subpleural infectious foci and interstitial lesions in his left lower lobe. He had traveled to Aba Prefecture 1 week earlier and had a smoking history of about 20 pack-years. An earlier CT scan suggested pulmonary bullae. His hypoxia symptoms progressed rapidly, and he was started on high-flow oxygen treatment, along with the antibiotics imipenem, moxifloxacin, and oseltamivir. The patient, however, quickly progressed to ARDS. Because mechanical ventilation with a 100% oxygen supply could not maintain saturation (IPPV, VT 480 ml; FIO2, 100%; PEEP, 14 cmH_2_O; f, 20/min; saturation, 70%), he was transferred to the ICU for ECMO treatment. Laboratory examination showed a white blood cell (WBC) count of 6.99 × 10^9^/L, a neutrophil rate of 91.7%, an aspartate transaminase concentration of 137.0 U/L, an alanine aminotransferase concentration of 25.0 U/L, a creatinine concentration of 414.0 μmol/L, a C-reactive protein (CRP) concentration of 259.39 mg/L, a procalcitonin (PCT) concentration of >200 ng/ml, a CD4+ lymphocyte count of 196/μl, and a CD8+ lymphocyte count of 27/μl. He was negative for β-(1,3)-glucan (BD), galactomannan, and influenza A and B. Chest CT showed multiple subpleural infectious lesions in the right lung, and interstitial changes in the lingual segment of the left upper lobe and left lower lobe (Figure 1A). The patient was treated with VV-ECMO at a flow rate of 3.8 L/min, a rotation speed of 7,400 rpm, and an airflow rate of 3.5 L/min. Later his blood and BALF mNGS showed only *Legionella pneumophila* infection (blood reads 229, BALF reads 656), and his antibiotic treatment was changed to moxifloxacin, azithromycin, and piperacillin-tazobactam sodium. Because of his acute kidney injury, ECMO was combined with continuous renal replacement therapy (CRRT). The patient's condition gradually improved, and he was weaned from ECMO on February 12 and from mechanical ventilation on February 13. A sputum smear showed no bacteria or fungi, sputum and blood cultures were negative, and he was negative for anti-*Legionella* antibodies. A comprehensive examination showed no evidence of immune system disease or tumor disease in this patient. A chest CT taken on February 19 showed significant improvement, and he was discharged to a local hospital for rehabilitation on February 20, 17 days after admission (Figure 1B). A blood test at that time showed a WBC count of 8.9 × 10^9^/L, a total neutrophil count of 6.42 × 10^9^/L, a neutrophil percentage of 72.2%, and a PCT concentration of 4.97 ng/ml.

**Figure 2:**
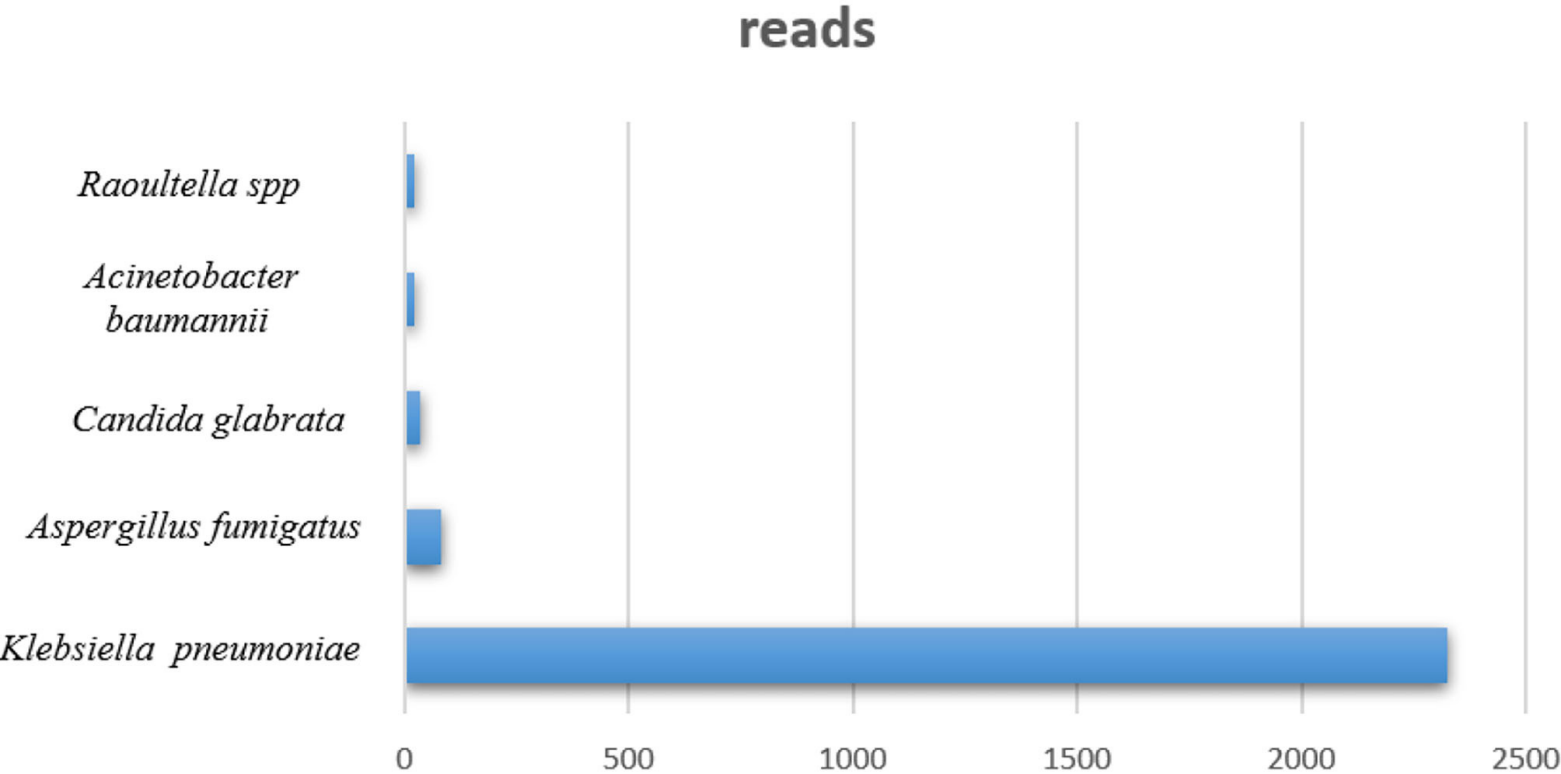
Detection of pathogens by mNGS.

**Figure 3:**
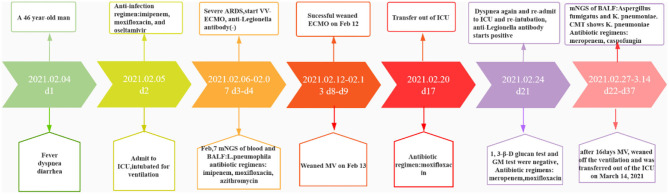
Course of treatment of this patient over time.

Four days after returning to the local hospital, the patient again presented with dyspnea, accompanied by chills and fever. Saturation could not be maintained by non-invasive positive pressure ventilation (NPPV). The patient was again intubated, and a large amount of bloody sputum was aspirated from his airways. The patient was re-admitted to the ICU, and a CT scan on February 24 showed scattered speckled shadows and reticular blurred shadows, with partial consolidation, consistent with infectious interstitial lesions (Figure 1C). Blood gas analysis showed pH 7.228, PO_2_ 7.3 kpa, PCO_2_ 9.64 Kpa, Lac 1.9 mmol/L, K^+^ 6.0 mmol/L, and 90% saturation (FIO_2_, 60%). The patient was started on treatment with meropenem plus moxifloxacin. He was positive for anti-*Legionella* IgM antibody, and his PCT was >200 ng/mL, but fungal BD and GM tests were negative. mNGS of bronchoalveolar lavage fluid (BALF) on February 27 revealed *Aspergillus fumigatus* and *Klebsiella pneumoniae*, with reads of 80 and 2,325, respectively (Figure 2). A culture of BALF in our hospital also suggested *K. pneumonia*. CT imaging showed bronchial wall thickening with local bronchiectasis and interstitial changes, and bronchoscopy showed airway erosion and congestion, resulting in a diagnosis of IPA and *K. pneumoniae* infection. Lung CT scan taken on March 1 indicated that the infection was still progressing (Figure 1D). After 16 days of mechanical ventilation, the patient was successfully weaned and transferred from the ICU on March 14. The treatment process is shown in Figure 3.

## Discussion

*Legionella* is a genus of aerobic gram-negative bacillus widely distributed in artificial cold water, natural water sources, moist soil, and hospital hot water systems. *Legionella* can be divided into 58 species with more than 70 different serotypes. *Legionella* pneumonia, which is mostly caused by *Legionella pneumophila* type 1, can progress rapidly to ARDS. The clinical diagnosis of *Legionella* is difficult, which may delay its treatment. At present, *Legionella* is diagnosed clinically by culturing by detecting antibodies in serum and antigens in urine, and by molecular detection techniques such as PCR; however, positivity rates are low (11). The patient described in this report, who had traveled elsewhere within 2 weeks prior to diagnosis, experienced an acute onset in the community and rapidly progressed to ARDS, combined with diarrhea and kidney damage. All these symptoms strongly suggested *Legionella* infection. Serum samples at the time of admission were negative for anti-*Legionella* antibody, and sputum cultures were negative. mNGS assays of BALF indicated *Legionella pneumophila*. *Legionella* grows in particular conditions, the positive culture rate is low, and more than 50% of early-stage patients have no sputum (4). Although urinary antigen tests are widely performed in clinical practice, these tests are sensitive only to *Legionella pneumophila* type 1. Moreover, urinary antigen tests were unavailable in our hospital. Metagenomic sequencing can significantly improve the early diagnosis rate of infectious diseases (12). Imaging studies of patients with clusters of onset, ineffective initial empirical treatment, severe community-acquired pneumonia, and travel history within 2 weeks before onset suggest that patients with multilobed lung lesions and bilateral pleural effusion should be routinely screened for *Legionella*.

Fluoroquinolones or macrolides are regarded as first line treatments for patients with *Legionella* pneumonia choice, with combination treatments recommended for critically ill or immunocompromised patients (13, 14). ECMO support has dramatically improved the survival rate of patients with severe *Legionella* pneumonia. For example, the survival rate of patients with *Legionella* pneumonia treated with ECMO was found to be 70% (15). Moreover, a single-center observational study of 112 ECMO-treated patients found that 12 of these patients had *Legionella* pneumonia, with a survival rate of 85.7% (16). The patient described in this report was weaned off ECMO after 6 days and recovered.

However, 12 days after ECMO withdrawal, the patient again presented with dyspnea and coughed up considerable bloody sputum. BALF culture indicated the presence of *K. pneumoniae*, and mNGS indicated *K. pneumoniae* and *A. fumigatus*. IPA occurs frequently in severely immunosuppressed patients, including organ transplant recipients, HIV-infected patients, patients with long-term or severe neutropenia, patients with tumors, and patients with long-term use of glucocorticoids. IPA, however, is a less well-recognized complication in patients without traditional risk factors. More recent reports have described patients with non-neutropenia-related IPA, including patients with end-stage chronic obstructive pulmonary disease (COPD) and Child-Pugh C cirrhosis, and patients receiving immunosuppressive therapy (17). Patients with severe influenza admitted to the ICU due to respiratory failure are also at risk for IPA, a condition called influenza-related pulmonary aspergillosis (18). Accidental IPA has been observed in about 1% of critically ill patients who undergo lung biopsy after death. Factors associated with IPA in critically ill, non-immunocompromised patients include COPD, ARDS, liver failure, and organ dysfunction, as well as treatment with glucocorticoids or broad-spectrum antibiotics (19).

The diagnosis of IPA remains challenging in non-immunosuppressed patients. The criteria for IPA caused by neutropenia may be inapplicable to non-neutropenic patients. mNGS can be used to assist in the diagnosis of IPA. For example, a comparison of mNGS with traditional microbial diagnostic methods for diagnosing *Aspergillus* found that mNGS had an accuracy of 78.3%, owing chiefly to the difficulty of extracting nucleic acids from molds, but a specificity as high as 97.5% (20). A search of PubMed identified only six case reports describing patients with secondary invasive pulmonary *Aspergillus* infection after *Legionella* infection (21–26) (Table 1). Of these six patients, one was diagnosed by autopsy, one by histopathology, and four by microbiological isolation and culture. The earliest diagnosis time was 6 days, but this patient eventually died. To our knowledge, this was the first patient with early diagnosis of *Legionella* and IPA through mNGS who was successfully treated with ECMO. *Legionella* infection has been reported to be a potential risk factor for IPA infection (14). The risk factors of IPA in this patient may have included admission to the ICU, the use of broad-spectrum antibiotics, and the placement of the ECMO circuit. The incidence of VV-ECMO complicated by IPA was 7%, the median duration of ECMO treatment before Aspergillus isolation was 5 days, and immunosuppression and influenza virus infection may be risk factors (10). Physicians should therefore be highly vigilant for the occurrence of IPA in patients with influenza virus infection, immunodeficiency, and *Legionella* infection, especially those receiving ECMO support. These patients should undergo early fungal screening to avoid poor outcomes.

**Table 1:** Patients diagnosed with invasive pulmonary aspergillosis associated with *Legionella* pneumophila.

**References**	**Underlying diseases**	**Immune status**	**Time from onset to diagnosis**	**Method of IPA diagnosis**	**Antifungal therapy**	**Outcome**
Jiva et al. (21)	Asthma	Prednisone 40 mg/day, 3 weeks	11 days	Autopsy	Amphotericin B	Death
Vergne et al. (23)	Smoking history	Normal	6 days	Anatomo-pathological examination	Amphotericin B	Death
Guillouzouic et al. (24)	Broad passive nicotinism	Prednisone 100 mg/day, 1 month	Not mentioned	*A. fumigatus* isolation from BALF	Caspofungin and voriconazole	Death
Saijo et al. (25)	Mild alcoholic liver injury	Normal	8 days	*A. fumigatus* isolation from BALF	Micafungin and voriconazole	Death
Coulon et al. (22)	Hypertension, type 2, diabetes, Smoking history	Prednisone 50 mg/day, 9 days	12 days	*A. fumigatus* isolation from BALF	Amphotericin B	Death
Shorten et al. (26)	Leukemia	Neutropenia	16 days	*A. fumigatus* isolation from peritoneal fluid	Voriconazole	Death
Present patient	Smoking history	Normal	3 days	mNGS	Caspofungin	Recovery

## Conclusions

The present study described a patient with severe *Legionella* pneumonia, diagnosed early by mNGS, who was found to have secondary IPA infection by mNGS. The patient was quickly diagnosed and successfully treated with ECMO and antibiotics. *Legionella* may enhance infection by invasive aspergillus. Patients infected with *Legionella* should undergo early complete screening of secondary infection using mNGS technology.

## Author Contributions

XH and LP were involved in the conception and design of the work. RY wrote the first draft of the manuscript and revised it. LP revised the manuscript. TL, XW, and LC were responsible for the management of this patient, for collecting the clinical data, and for reviewing the literature throughout this study. All authors contributed to the article and approved the submitted version.

## Conflict of Interest

The authors declare that the research was conducted in the absence of any commercial or financial relationships that could be construed as a potential conflict of interest.
